# Michigan Dental Association

**Published:** 1862-01

**Authors:** J. A. Harris

**Affiliations:** Pontiac


### Proceedings of Societies.

### MICHIGAN DENTAL ASSOCIATION.

Detroit, Jan. 7th, 1862.

The seventh annual meeting of the Michigan Dental Association was held at the Biddle House, in the city of Detroit, on the 7th, at 7 o'clock, P. M., President, Dr. Wm. Cahoon, in the chair.

The Minutes of the last session were read.

The election of officers postponed until to-morrow.

The Association adjourned until 9, A. M., Wednesday.

MORNING SESSION.

Association met pursuant to adjournment, 9 A M., when regular order of business was called by President.

Committee on order of business reported—

1. Reading Minutes of preceding meeting.
2. Reception of reports from officers and committees.
3. Election of members.
4. Election of officers.
5. President's address.
6. Unfinished business.
7. Reading essays and papers, and remarks on same.
8. Payment of dues.
9. Miscellaneous business.

The report was adopted.

The Secretary made his annual report. Adopted.

The Treasurer being absent, there was no report.

The following officers were elected for the ensuing year, to-wit:

*President.*—Dr. J. A. ROBINSON, of Jackson.

*Vice-President.*—Dr. J. DOUGLASS, of Romeo.

*Secretary.*—Dr. J. A. HARRIS, of Pontiac.

*Treasurer.*—Dr. H. BENEDICT, of Detroit.

After a few remarks from the retiring President, Dr. Robinson was conducted to the chair, thanking the Association for the honor they had conferred upon him, hoping his intercourse with them would be as pleasant in the position he now occupied, as it had been in the former.

The Vice-President then took the chair, when

Dr. ROBINSON read an interesting essay on "Plugging teeth," which was ordered to be placed upon the records of the Association, and published. The essay elicited a general discussion upon the merits of the different materials used for plugging, especially of gold foils.

Dr. CAHOON spoke at some length, stating his method, and experience in using foils of different manufactures ; uses both soft and adhesive, but preferred soft, of Abbey's manufacture; also uses White's and Dunlevy's, annealing always before using, the adhesion being perfect, making a solid plug, and building out to any degree desired; had observed the great difficulty with many operators was, they cut up the gold after placing in cavity ; that it becomes entirely disintegrated, or in other words, pulverized, losing its adhesive properties, and becoming solidified. His method was to work gently at first, until adhesion was perfect, then condense.

Dr. METCALF wished to know whether the adhesion between soft and crystal foil was perfect. His experience in the South, where he had practiced extensively, did not prove to him that it was; thinks it must be owing to dampness of the atmosphere, but by annealing previously to using, had no difficulty —uses pellets, always anchors, or fastened the first.

Dr. ROBINSON says, in all his experience in building out front teeth with crystal and Abbey's soft foil No. 4, the union was perfect, and he was daily convinced of the fact, by seeing many cases of his own practice, that had stood the test of years, and he was rejoiced to see the profession daily and yearly rising to occupy its appointed place, which in his mind stood second to none.

Dr. BENEDICT wished te make a few remarks on the essay, in the shape of criticism, in regard to his (Robinson's) method of plugging teeth.

Dr. ROBINSON appealed to the paper to show his meaning, which was declared satisfactory.

Dr. B. uses both soft foil and adhesive, using the soft at first, finishing with adhesive, the secret of success being in the care of the operator in fingering his foil. You may be as cautious as possible ; it will gather moisture from the insensible perspiration of the fingers, though the foil should always be annealed before using, to dry this moisture.

Dr. WHITE read an essay on "Treatment of diseased nerves and fangs."

In the remarks that followed, it was thought that too much arsenic was used; that after treatment, the tooth must be entirely well, devoid of all soreness either externally or internally, the nerve removed to the apex, and healthy granulations found; that unless these results had taken place, the chances were against the success of the operation. In destroying nerve, always make the nerve bleed, or uncap it by removing the soft decay, and in nine cases in ten, the pain will cease. You may place creosote, or whatever is used in cavity without, and it will surely give the patient much pain, and upon his return, you will have to do what ought to have been done at first.

The Association then took a recess to 2, P. M.

2 O'CLOCK, P. M.

Dr. CAHOON read an essay on "Dentistry as a Profession," which was ordered to be placed on record, and published.

In remarks upon the essay, the subject of fees was discussed warmly, especially of prices of vulcanite, it being contended that a severe blow had been struck the profession in this regard, although it was held to be vastly superior to a majority of materials used for dentures. A general feeling was manifested that the prices must be kept up, or the profession go down.

Dr. CAHOON was in favor of putting the price of rubber to its former mark, and earnestly hoped the Association would take some action on the subject. The public had been benefited by the improvements of the profession, and the dentist should have a fair remuneration for his services.

Dr. ROBINSON did not think we ought to be extortionate, but was in favor of keeping up the prices; was of the opinion that rubber would last equally as well as gold.

Dr. BENEDICT liked rubber, but did not make much of it, as he did not wish to compete with those that were making it at mechanic wages. Many were using it without having purchased a license, and were evidently trying to see how cheap they could make it.

A letter was read from Dr. BARTLETT, of Battle Creek, which was ordered placed on file, when the Association took recess, after which the following resolutions were offered by Dr. Robinson:

*Resolved*, That it is unprofessional and ungentlemanly for any member of this Association to lease his place of business, and visit neighboring towns, for the purpose of getting work, where there is a resident dentist, belonging to this Association, unless particularly solicited.

On motion,

*Resolved*, That the Secretary issue a warrant on the Treasurer, to defray the expense of room, etc.

*Resolved* That the Secretary also issue a warrant on the Treasurer, of six dollars, to balance the amount due Dr. Cahoon.

*Resolved*, That a vote of thanks to the proprietors of the Biddle House, for the use of their room, and their gentlemanly bearing to the members of the Association, be given.

All of which were adopted.

The following gentlemen were appointed to prepare essays on the subjects assigned them;

| | |
|---|---|
| Dr. Douglass, | Diseased Antrum. |
| " White, | Vulcanite. |
| " Mansfield, | Treatment of exposed nerve. |
| " Benedict, | Dental Fees and Etiquette. |
| " Metcalf, | Filling Teeth, |
| " Bancroft, | Alveolar Abscess. |
| " Harris, | Extracting Teeth. |
| " Porter, | Taking Impressions. |
| " Cahoon, | Best Material for Artificial Dentures. |
| " Robinson, | History of Dentistry. |

Moved, That we now adjourn to meet in the city of Kalamazoo, on Tuesday, the 27th of January, 1863, at 9 o'clock, A. M.

All were pleased to see Dr. S. D. Palmer, who was on hand as usual, with a large supply of teeth, instruments, etc. Dr. Pritchard, of Rochester, was also present.

J. A. HARRIS, Pontiac, *Cor. Sec.*

